# Mediterranean Diet Adherence Is Associated with Favorable Health-Related Quality of Life, Physical Activity, and Sleep Quality in a Community-Dwelling Greek Older Population

**DOI:** 10.3390/antiox12050983

**Published:** 2023-04-22

**Authors:** Maria Mantzorou, Maria Mentzelou, Georgios K. Vasios, Christos Kontogiorgis, Georgios Antasouras, Konstantinos Vadikolias, Evmorfia Psara, Theofanis Vorvolakos, Efthymios Poulios, Aspasia Serdari, Sousana K. Papadopoulou, Constantinos Giaginis

**Affiliations:** 1 Department of Food Science and Nutrition, School of Environment, University of Aegean, 81400 Lemnos, Greece; mantzorou.m@aegean.gr (M.M.); fnsd22007@fns.aegean.gr (M.M.); vasios@aegean.gr (G.K.V.); fnsd22001@fns.aegean.gr (G.A.); fnsd21013@fns.aegean.gr (E.P.); epoulios@aegean.gr (E.P.); 2 Laboratory of Hygiene and Environmental Protection, Democritus University of Thrace, 68100 Alexandroupolis, Greece; ckontogi@med.duth.gr; 3 Department of Neurology, School of Medicine, Democritus University of Thrace, 68100 Alexadroupolis, Greece; kvadikol@med.duth.gr; 4 Department of Geriatric Psychiatry, School of Medicine, Democritus University of Thrace, 68100 Alexadroupolis, Greece; tvorvola@med.duth.gr; 5 Department of Psychiatry and Child Psychiatry, School of Medicine, Democritus University of Thrace, 68100 Alexadroupolis, Greece; aserntar@med.duth.gr; 6 Department of Nutritional Sciences and Dietetics, School of Health Sciences, International Hellenic University, 57400 Thessaloniki, Greece; sousana@the.ihu.gr

**Keywords:** Mediterranean diet, health-related quality of life, physical activity, sleep quality, public health, elderly, body mass index, antioxidant, anti-inflammatory, mental health

## Abstract

Background: The Mediterranean diet (MD) is a beneficial dietary pattern with strong antioxidant and anti-inflammatory properties that can promote mental and physical human health. This study aims to assess the impact of MD adherence on health-related quality of life, physical activity levels, and sleep quality in a representative Greek elderly population. Methods: This is a cross-sectional study. A total of 3254 persons ≥65 years from 14 different Greek regions, urban, rural and islands participated in this study, of which 48.4% were female and 51.6% were male. Health-Related Quality of Life (HRQOL) was evaluated utilizing a short form healthy survey, physical activity was determined by the International Physical Activity Questionnaire (IPAQ), sleep quality was assessed utilizing the Pittsburgh Sleep Quality Index (PSQI) and MD adherence was assessed via the Mediterranean Diet Score (MedDietScore). Results: Moderate adherence to the MD and an increased prevalence of poor quality of life, low physical activity levels and inadequate sleep quality among the elderly population were recorded. High MD adherence was independently associated with better quality of life (OR: 2.31, 95% CI: 2.06–2.68, *p* = 0.0008), higher physical activity (OR: 1.89, 95% CI: 1.47–2.35, *p* = 0.0141) and adequate sleep quality (OR: 2.11, 95%: 1.79–2.44, *p* = 0.0018), female sex (OR: 1.36, 95% CI: 1.02–1.68, *p* = 0.0032) and living with others (OR: 1.24, 95% CI: 0.81–1.76, *p* = 0.0375), after adjustment for potential confounding factors. In unadjusted analysis, participants’ age (*p* < 0.0001), anthropometric characteristics (*p* < 0.005), educational (*p* = 0.0026) and financial status (*p* = 0.0005) and smoking habits (*p* = 0.0031) were also identified as indicators of MD adherence; however, their impact on MD adherence was considerably attenuated after adjusting for confounding factors (*p* > 0.05). Conclusion: High MD adherence was correlated with favorable quality of life, higher levels of physical activity, and a more adequate sleep quality score. Strategies and public health policies that facilitate MD adherence and physical activity in older adults may improve sleep and quality of life, impacting overall wellbeing in this age group.

## 1. Introduction

The global population is ageing, affecting individuals and societies. Healthy ageing is a global priority, as we are going through the United Nations Decade of Healthy Ageing (2021–2030) [1]. Key health-related and well-being factors for healthy ageing have been associated with a healthy lifestyle that includes adherence to a healthy dietary pattern and a physically active lifestyle. The Mediterranean diet (MD) is linked with nutritional patterns that are characteristic of specific areas of Greece and Italy from the first years of 1960s, in which adult life expectancy was considerably elevated and the prevalence of nutrition-associated chronic disorders was decreased [1,2]. High MD adherence has been related with a lowered probability of various chronic disorders [2], especially cardiovascular disease [3], cancer [4], and type 2 diabetes mellitus [5], as well as cognitive decline and depression in older adults [6]. In fact, adherence to the MD has a key role in healthy ageing [7], which is also related to greater physical health and health-related quality of life as well as several aspects of lifestyle related to social attitudes, cognitive functioning, mental health, daily physical activity and well-being and clinical characteristics [8]. Ageing is a natural process that has been linked with oxidative stress (e.g., oxidative DNA damage, DNA repair systems’ inhibition, reactive oxygen species production, telomere attrition, epigenetic alterations, etc.) and inflammatory cascades (e.g., proinflammatory mediators’ inhibition, malondialdehyde and superoxide dismutase decreases, cyclooxygenases’ inhibition, platelets and white blood cells count decreases, etc.) [9,10]. The above favorable effects of MD have primarily been ascribed to its food components, such as fruits, legumes, vegetables, olive oil, herbs, spices, and fibers, which are rich in numerous bioactive compounds, with a plethora of antioxidant and anti-inflammatory properties [11,12]. Moderate wine consumption when included in an MD model has also been associated with reduced risk of chronic degenerative diseases due to its antioxidant constituents [13,14]. The MD is not limited to a strict healthy dietary pattern, but it includes specific skills, knowledge, rituals, symbols, and traditions regarding crops, harvesting, fishing, animal husbandry, conservation, processing, cooking, and especially the sharing and consumption of foodstuffs [13,14]. Eating with others constitutes the basis of the cultural identity and continuity of societies in the Mediterranean region. It is a moment of social exchange and connection, and a confirmation and regeneration of family, group, or societal identity [13,14]. Moreover, in the traditional MD model, a significant amount and type of foods consumed were local and home-made/grown, especially in the context of dairy products, olive oil, and wine [13,14].

The concept of MD comprises physical activity via implementing daily routines and a robust sense of community. These aspects of the lifestyle are not directly related with diet per se, but do enhance the Mediterranean paradigm as a lifestyle which supports human health in a holistic manner. In this context, MD is a beneficial dietary pattern for both physical and mental health, even in older adults with depression [15]. In older adults, an age group at high risk of mental diseases and depression [6], MD adherence leads to mixed results regarding quality of life [16]. In fact, an analysis of two cohorts in Spain failed to find an association between MD and mental health, even though one of the cohorts did find that the PREDIMED score correlated with the physical component of the health-related quality of life (HRQOL) score [17]. Other studies found significant associations between MD adherence and quality of life, and physical and mental health status in older persons [18,19]. The different conclusions of the above studies may be ascribed to the use of different questionnaires regarding MD and quality of life, as well as different populations (Spain and North America, respectively) and different age groups (>60 years old and 45–79 years old, respectively) [18,19]. The antioxidant and anti-inflammatory effects of the MD overall and the effects of specific MD constituents, especially extra virgin olive oil, fruits and vegetables, whole grains, and fish, may account for the favorable findings on physical health and wellbeing [13,14,15]. In addition, wine contains a plethora of phytochemicals (like fruits and olive oil), and is assumed to counteract oxidative stress and promote antiatherogenic actions through its high polyphenolic content [13,14,15].

World Health Organization standards propose that older adults must engage in at least “150–300 min of moderate-intensity aerobic physical activity” “as part of their weekly physical activity” [20]. In fact, a recent study emphasized that older adults are not even aware of these exercise guidelines [21]. Physical activity seems to be associated with greater physical and mental health status and favorable quality of life in older adults, as well as longevity [21,22,23]. Frequent physical activity is suggested because of its health benefits concerning cardiometabolic health, excess body weight, cognitive function, and musculoskeletal health [24]. Nevertheless, physical activity levels in this age group are low, and result in health implications [25] and elevated risk of mortality [26]. More to the point, physical activity may improve depression symptomatology and quality of life in institutionalized older individuals [27] and prevent falls in this age group [28].

Moreover, older adults are characterized by a high probability of sleep disturbances [29,30]. Long and short sleep may result in chronic disease, with data indicating that short sleep duration could raise the likelihood of type 2 diabetes mellitus and glucose metabolism dysregulation, as well as cardiovascular disease [31,32,33,34]. Insufficient sleep may also reduce dietary efforts to reduce adiposity [35] and maintain a healthier body weight [33,34,35,36,37]. Moreover, there is an interrelationship between diet and sleep, and greater MD adherence seems to promote adequate sleep duration, being also associated with various markers of favorable sleep quality [37,38]. Micronutrient deficiencies, and especially inadequate intake of calcium, magnesium, and vitamins A, C, D, E, and K, may increase the risk of short sleep duration [39]. In older adults with insomnia, micronutrient supplementation with melatonin, magnesium, and zinc improved both sleep quality and sleep duration [40], while tart cherry juice improved insomnia severity, and fermented milk improved sleep efficiency [41,42]. Certain data suggest that a higher MD adherence may improve quality of life, physical activity, and sleep quality; however, the existing findings still remain limited in older populations in Greece [42].

In this aspect, the present cross-sectional survey intends to investigate the relationships between MD adherence and health-related quality of life, physical activity levels, and sleep quality in a community-dwelling Greek elderly population from 14 distinct areas, including urban, rural and island regions. There is a lack of evidence in our country, as well as in Mediterranean countries, and thus we performed this study to cover this international literature gap.

## 2. Methods

### 2.1. Study Population

At first, 6482 community-dwelling Greek Caucasian older persons over 65 years old were randomly assigned from 14 distinct, geographically diverse Greek areas, both urban, rural and islands, namely Athens, Thessaloniki, Alexandroupoli, Kavala, Ioannina, Larissa, Lamia, Korinthos, Patra, Tripoli, Kalamata, Crete, North and South Aegean. Recruitment to the study was from April 2014 to December 2019 in community-dwelling older persons who were principally enrolled during their visits to health care units, as well as to public centers dedicated to entertainment activities for older individuals.

During their careful enrollment, older persons who suffered from serious chronic disease symptomatology (*n* = 3228), including any cardiovascular diseases, any cancer or premalignant disease, metabolic disturbances, autoimmune disorders, or neurodegenerative diseases, were excluded from the study. In the end, 3254 older persons were enrolled in the study, leading to a final response rate of 50.2%.

All information of the enrolled older adults was confidential. All enrolled older adults were informed about the aim of the study, and signed a consent form. The guidelines of the Declaration of Helsinki were applied according to the World Health Organization (52nd WMA General Assembly, Edinburgh, Scotland, 2000). The Ethics Committee of the University of Aegean (ethics approval code: no 10/24.3.2014) approved the design and the implementation of the current study, as well as the consent approval of the enrolled older adults.

### 2.2. Study Design

Three well-recognized questionnaires were utilized to assess health-related quality of life, physical activity levels and sleep quality of the older adult participants. More to the point, Health-Related Quality of Life (HRQOL) was determined by using the Short Form Healthy Survey (SF-36) questionnaire, which contains 36 items evaluating health status within 8 subcategories [43]. The first 4 subcategories evaluate physical HRQOL: physical functioning, physical role limitations, bodily pain, and general health perception. The next 4 subcategories evaluate mental HRQOL: role limitations because of emotional troubles, vitality, mental health, and social functioning. For those subcategories, a score ranging from 0 (worst) to 100 (best) is estimated. All but one item are assigned to one of the 8 health domains covering diverse features of physical and mental health: physical functioning (PF, 10 items), physical role functioning (RP, 4 items), bodily pain (BP, 2 items), general health perceptions (GH, 5 items), vitality, (VT, 4 items), social role functioning (SF, 2 items), emotional role functioning (ER, 3 items), and mental health (MH, 5 items) [43].

We assessed physical activity levels utilizing the International Physical Activity Questionnaire (IPAQ) that determines how much exercise the responders engage in during a usual week. This self-administered questionnaire evaluates all physical activity engaged in over the previous 7 days, to classify it as low, moderate, or high [44]. IPAQ instruments have thoroughly been assessed worldwide, being characterized by sufficient consistency and adequate validity, at least as good as other self-reported PAQs. In brief, the aim of IPAQ-Gr is to sum up vigorous, moderate, and walking PAs during the last 7 day period and produce an overall physical activity score (PAscore), quantified by MET minutes per week (MET.min.wk-1). According to the IPAQ scoring process, PA status is categorized into 3 classes (PAclasses): (1) low PAclass, inadequately active subjects (total PAscore < 600 MET.min.wk-1); (2) modest PAclass; and (3) great PAclass, HEPA active subjects, (HEPA: health-enhancing physical activity, i.e., total PAscore ≥ 3000 MET.min.wk-1 or vigorous PAscore ≥ 1500 MET.min.wk-1 [44]).

We further assessed sleep quality utilizing the Pittsburgh Sleep Quality Index (PSQI), which includes 19 items rated on a 4-point scale (0–3) and clustered into 7 components (sleep quality, sleep latency, sleep duration, habitual sleep efficiency, sleep disturbance, use of sleeping medications, and daytime dysfunction). The item scores in each component were summated and converted to component scores ranging from 0 (better) to 3 (worse) according to the guidelines [45]. Overall PSQI scores were estimated by the sum of the 7 component scores, ranging from 0 to 21, where higher scores show a worse condition. A total global PSQI score of <5 is indicative of adequate sleep quality guidelines [45].

For the evaluation of the MD compliance, we used the Mediterranean Diet Score (Med-DietScore) by Panagiotakos et al. [46]. This is a Food Frequency Questionnaire (FFQ) with 11 chosen food patterns according to Med Diet Score index [46]. Each question exhibits 6 potential responses, rated from 0 to 5, depending on the degree of compliance for each specific food pattern. The summation of the 11 questions results in a score from 0 to 55; a higher score reflects greater MD compliance. For cereals, potatoes, fruits, vegetables, dairies and olive oil, a scale of 6 potential answers was adopted per day. For legumes, seafood, red meat and poultry, a scale of 6 potential responses was adopted per week [46]. The 11th question assessed wine consumption per day, with moderate consumption (≤1 and ≤2 drinks/day for women and men, respectively (one drink = 100 mL = 12 g ethanol)) taking the highest score [46].

Qualified medical and nursing personnel, nutritionists and dietitians comprehensively described the above questions to the community-dwelling elderly to obtain reliable responses. A face-to-face interview was conducted, in which each interviewer promptly connected with each participant in accordance with the planned questionnaires. Qualified personnel measured anthropometric indices, body mass index (BMI), mid arm circumference and calf circumference as per protocol [6,47]. We also assessed education level based on years of education. Sample size estimation was performed using the PS: Power and Sample Size calculator program, while a simple randomization method was applied using a sequence of random binary numbers (e.g., 010101110 in which 0 represented enrolment and 1 not enrolment to the study). PS software can estimate the sample size recommended to obtain a specified alternate hypothesis with the required power, i.e., the power with which a specific alternate hypothesis can be obtained with a given power and sample size. We measured the body weight of the participants utilizing the same electronic scale, as well as participants’ height utilizing a portable stadiometer (Chapter HM200P, Medi Shop, Greece). We also measured mid-arm and calf circumference, which are indicators of muscle mass, using a non-elastic measuring tape [6,47].

We assessed economic level, living status and smoking habits based on the answers of the participants. Financial status was categorized based on the annual income as: 0 ≤ 5000 EUR, 1 ≤ 10,000 EUR, 2 ≤ 15,000 EUR, 3 ≤ 20,000 EUR, 4 ≤ 25,000 EUR and 5 > 25,000 EUR, according to per capita gross domestic product. We classified financial status as low for annual income ≤ 10,000 EUR, medium for annual income >10,000 EUR and ≤ 20,000 EUR, and high for annual income > 20,000 EUR. We also classified living status as living completely alone or living with others, i.e., a husband or wife, their children, or other relatives. We also recorded smoking habits, classifying the participants’ older adults as never smokers or smokers.

### 2.3. Statistical Analysis

We used a Student’s *t*-test and a one-way ANOVA for continuous variables that followed the normal distribution, using a Kolmogorov–Smirnov test. We applied a Chi-square test for categorical variables. The normally distributed quantitative variables are presented as mean value ± standard deviation (SD), and the qualitative variables as absolute or relative frequencies. We performed multiple logistic regression to evaluate the effect of MD adherence on health-related quality of life, physical activity levels and sleep quality by adjusting for potential confounding factors. As confounding factors, we included all the available co-variates, since all of them may exert a confounding effect. Multiple regression results are stated as odds ratios (OR) and 95% confidence intervals (CI). Differences were considered significant at *p* < 0.05 and 95% CI. Statistica 10.0 software, Europe (Informer Technologies, Inc., Hamburg, Germany) was applied for the statistical analysis of the survey data.

## 3. Results

### 3.1. Sociodemographic and Anthropometric Characteristics and MD Adherence Evaluation

The mean age of the older adult participants was 74.7 ± 8.4 (range: 65–88 years old) years old. Regarding sex, 48.4% were women and 51.6% were men. BMI measurements followed normal distribution and their mean value was 27.3 ± 4.1 (range: 17.2–41.0). Some 74.1% of older adults had a mid arm circumference ≥ 22 cm, and 25.9% of the elderly had a mid arm circumference < 22 cm (mean: 24.2 cm, range: 19.8–28.7 cm). A total of 64.4% of participants had a calf circumference ≥ 31 cm, and 35.6% of older adults had a calf circumference < 31 cm (mean 33.2 cm, range: 28.3–37.2 cm). The mean value of the educational years was 7.3 ± 2.8 years (range: 0–14 years).

MD adherence was assessed by MedDietScore. MedDietScore variable followed normal distribution based on Kolmogorov–Smirnov test with a mean value of 28.3 ± 4.2 points (range: 11–42 points) and an interquartile range (IQR) between 25 and 31 points. Participants were divided into quartiles based on MedDietScore [6,8,46,47]. Older adults with score ≤ 25 (25.8%) were categorized as presenting “very low” MD compliance, while participants with scores ranging from 26 to 28 (25.6%) were classified as presenting “low” MD compliance. Older adults with scores ranging from 29 to 31 (24.8%) were categorized as presenting “moderate” MD compliance, while participants with a score ≥ 32 (23.8%) were classified as presenting “high” MD compliance [6,8,46,47].

As far as financial status is concerned, 42.5% of the older adults stated low annual income, 38.2% medium and 19.3% high annual income. As far as living status is concerned, 36.5% of the older adults were living alone and 63.5% were living with others. Regarding smoking habits, 25.4% were smokers and 74.6% were never smokers.

### 3.2. Health-Related Quality of Life, Physical Activity, and Sleep Quality Evaluation

As far as health-related quality of life is concerned, based on the HRQOL questionnaire, the median score was 55.3/100 (IQR: 38.2–74.2). HRQOL was categorized into quartiles; 25.1% of participants had very low health-related quality of life, 24.0% had low health-related quality of life, 26.2% showed moderate quality of life, and the remaining 24.7% had high HRQOL scores. Considering the physical activity levels of the study population based on IPAQ classification, 40.9% had low physical activity levels, 35.3% had moderate physical activity levels and 23.8% had high physical activity levels. Concerning the sleep quality of the participants, based on PSQI classification, 55.6% of older adults had adequate sleep quality and 44.4% had inadequate sleep quality.

### 3.3. Association of MD Adherence with Sociodemographic and Anthropometric Characteristics

In crosstabulation, MD adherence was significantly higher in younger compared to older participants (Table 1, *p* < 0.0001). Higher MD adherence was more frequently observed in female than male participants (Table 1, *p* < 0.0001). MD adherence was negatively associated with elderly BMI (Table 1, *p* = 0.0047). The proportion of older adults with a mid arm circumference < 22 cm, an indication of low muscle mass, was gradually reduced from moderate/high to very low/low MD compliance (Table 1, *p* = 0.0008). Moreover, the prevalence of older adults with a calf circumference < 31 cm was progressively decreased from very low/low to moderate/high MD compliance (Table 1, *p* = 0.0011).

Educational level and economic status were positively associated with MD adherence (Table 1, *p* = 0.0026). Older adults living with others had significantly higher rates of moderate/high MD adherence than those living alone (Table 1, *p* = 0.0016). Never smokers showed a significantly higher prevalence of moderate/high MD compliance than smokers (Table 1, *p* = 0.0031).

### 3.4. Association of MD Adherence with Health-Related Quality of Life, Physical Activity, and Sleep Quality

In crosstabulation, higher MD compliance was considerably related with better HRQOL (Table 1, *p* < 0.0001). Older adults with moderate or high MD adherence had substantially more frequently high physical activity levels than those with low or very low MD adherence (Table 1, *p* < 0.0001). Older adults with moderate or high MD compliance had considerably more frequently adequate sleep quality than those with low or very low MD compliance (Table 1, *p* < 0.0001).

**Figure 1:**
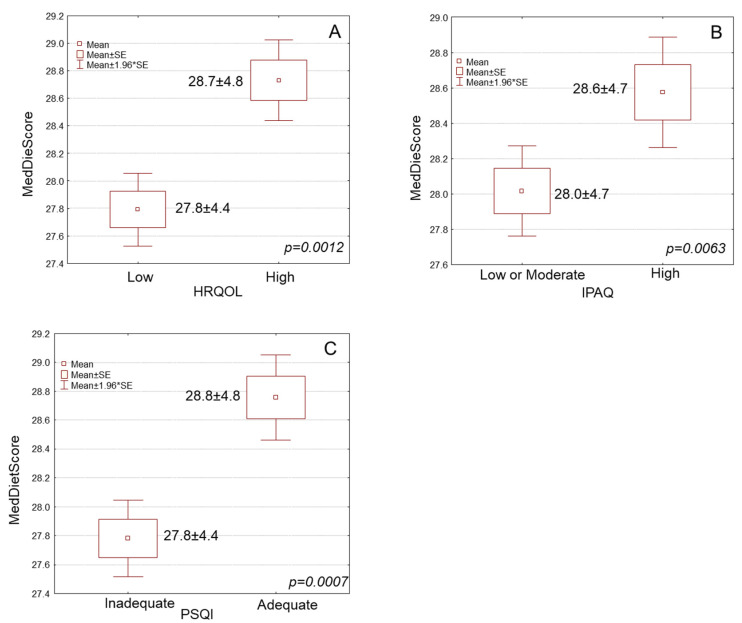
Box and whisker plots for Mediterranean diet adherence (MedDietScore) of elderly participants in association with their (**A**) HRQOL, (**B**) IPAQ and (**C**) PSQI. HRQOL: Health-Related Quality Of Life, IPAQ: International Physical Activity Questionnaire, PSQI: Pittsburgh Sleep Quality Index, SE: Standard Error, *: by.

Using MD adherence as continuous variable, older adults with high HRQOL had a significantly higher mean MedDietScore than those with low HRQOL (Figure 1A, 28.7 ± 4.8 vs. 27.8 ± 4.4). Participants with high physical activity levels had a considerably greater mean MedDietScore than those with low or moderate physical activity levels (Figure 1B, 28.6 ± 4.7 vs. 28.0 ± 4.7). Participating individuals with adequate sleep quality had a significantly higher mean MedDietScore than those with inadequate sleep quality (Figure 1C, 28.8 ± 4.8 vs. 27.8 ± 4.4).

### 3.5. Multiple Regression Analysis for MD Adherence after Adjustment for Potential Confounding Factors

In multiple logistic regression analysis, MD adherence was independently related with the participants’ sex, living status, HRQOL, physical activity and sleep quality, after adjustment for possible confounders (Table 2). Specifically, female older adults had 36% higher odds of higher MD adherence than male elderly (Table 2, OR: 1.36, 95% CI: 1.02–1.68, *p* = 0.0032). Older adults living with others had a 24% greater incidence of higher MD adherence than those living alone (Table 2, OR: 1.24, 95% CI: 0.81–1.76, *p* = 0.0375). Older adults with moderate or high MD adherence had more than 2-fold better HRQOL levels than those with very low or low MD adherence (Table 2, OR: 2.31, 95% CI: 2.06–2.68, *p* = 0.0008). Moreover, older individuals with moderate or high MD adherence had 89% higher odds of better physical activity levels than those with very low or low MD adherence (Table 2, OR: 1.89, 95% CI: 1.47–2.35, *p* = 0.0141). Older adults with moderate or high MD adherence had a 2.1-fold better sleep quality than those with very low or low MD adherence (Table 2, OR: 2.11, 95%: 1.79–2.44, *p* = 0.0018). The participants’ age, BMI, mid-arm and calf circumference, educational and financial levels and smoking habits did not remain significant in the multiple regression analysis (Table 2, *p* > 0.05).

## 4. Discussion

In our study, higher MD adherence was independently associated with better quality of life, greater physical activity levels and more adequate sleep quality. Participants’ age, and gender, anthropometric and sociodemographic factors and certain aspects of lifestyle were also identified as indicators of MD adherence; however, their impact on MD adherence was considerably attenuated after adjustment for confounding factors. We recorded lower mean MedDietScore scores within the older adult population affected by high rates of poor quality of life, low physical activity levels and inadequate sleep quality. The results of our study agree with the findings of other relevant studies. In terms of adherence to the MD, the Greek population [48] as well as other Mediterranean populations are moving away from the traditional dietary patterns [44], prompting negative impacts on health status and longevity [45].

In accordance with our findings, a plethora of studies around the world in older adults, both men and women, have also highlighted the positive association between MD and HRQOL, despite using different questionnaires [49,50,51,52,53,54]. The SUN cohort with 11,015 participants and a 4-year follow-up found an independent association between MD compliance and all the physical and many of the mental health domains of the SF-36 questionnaire [55]. The SUN cohort also showed that the “Western” diet has a negative effect on quality of life, as assessed by the SF-36 questionnaire after a 4-year follow-up [56]. Furthermore, the MARK study, in people with intermediate cardiovascular risk, found that greater diet quality and adherence to the MD pattern seem to be linked to better quality of life [57]. In older populations, a higher HRQOL score appears to be linked to a better nutritional status, good sleep quality, living with family members, closer social and family connections and better health status [58].

Sleep quality in association with dietary patterns has not been adequately investigated in the international literature, especially in older populations. Among the older adults, poor sleep quality seems to be a risk factor for obesity, hypertension, metabolic syndrome, and type 2 diabetes mellitus, which are also related to diet quality [59,60,61]. Sleep quality is multi-factorial, and the mechanisms behind the associations between nutrients, dietary patterns and sleep are not yet clear. The effect of the MD on sleep appears to be influenced by the effects of its characteristic foods and nutrients [62]. In line with our findings, several studies have provided evidence that supports that the MD may improve sleep duration and/or sleep quality [31] throughout life [63,64,65,66], as well as during pregnancy [67].

In accordance with our results, Mamalaki et al. also found that high MD compliance was independently related with better sleep quality in a Greek elderly population based on the sleep scale from the Medical Outcomes Study, even if MD adherence was not associated with sleep duration [65]. Campanini et al. also found that higher adherence to the MD improved sleep quality both in terms of sleep duration changes and sleep quality, based on Epworth Sleepiness Scale [66]. In fact, in middle aged adults, a lower PSQI score resulted in lower MD adherence and higher BMI, implying that interventions in diet could potentially favor sleep and body weight [68]. Accordingly, in women aged 20–76 years old, a higher alternate MD score improved sleep quality based on PSQI scores [69]. The positive impact of the MD on insomnia has also been described in a study by Castro-Diehl et al., in which people with a higher MD adherence score had a lower risk of insomnia and short sleep, while over a 10-year follow-up, stable MD adherence levels resulted in fewer insomnia symptoms, compared to lower MD adherence levels [70].

Moreover, our findings regarding sleep quality are in line with other studies that have found positive associations between diet and sleep quality, as evaluated by self-reported or objective methods [71]. A cross-sectional study in 810 very old adults over the age of 85 years showed that those with a “good” self-reported sleep quality had a “moderate-to-high” diet quality, even after adjustment for several confounding factors [72]. Another paper by Godos et al. [73], as part of the Mediterranean healthy Eating, Ageing, and Lifestyle (MEAL) study, documented that a diet with a higher Dietary Inflammatory Index (DII^®^) brought a higher risk of inadequate sleep quality in adults, as assessed by the PSQI questionnaire, while Rostami et al., using the same sleep quality questionnaire, recently found that adherence to the Mediterranean-DASH Intervention for Neurodegenerative Delay (MIND) Diet brought a lower risk of poor sleep quality in adults [74].

A recent review by Sejbuk et al. focused on the interconnected associations between diet, physical activity, and sleep, and concluded that several factors may impact sleep, such as anxiety and genes, nutrition, physical activity, and sleep hygiene [75]. In fact, low protein intake [76] and low-in-fiber carbohydrates [77] can negatively impact sleep. Additionally, the frequent consumption of noodles, sweets, and sugar-sweetened drinks, skipping breakfast, and not having routine meals promoted worse sleep quality. On the other hand, a dietary pattern high in fish, seafood and vegetables considerably improved sleep quality [77,78]. 

A healthy and active lifestyle according to the MD lifestyle pattern has been very well documented as a means of healthy ageing. In our study, the older adults that had a higher MD adherence were also more physically active. In this aspect, the MD and its food components can positively affect ageing, lowering the risk of chronic disease and physical impairment, while also preventing frailty [79,80]. In this regard, a recent systematic review and meta-analysis on 19 cross-sectional studies with 19,734 older adults found that higher MD adherence can considerably improve physical performance tests scores [81].

Moreover, both the adherence to a healthy dietary pattern and a physically active lifestyle may promote physical and mental wellbeing and longevity [82,83]. Regarding the role of lifestyle, the HALE project did find that during a 10-year follow-up, older adults aged 70–90 years who had a good adherence to the MD, were non-smokers or did not smoke for more than 15 years, were physically active, and drank alcohol in moderation had less than half the mortality rate from all causes, highlighting the important role of lifestyle in ageing and early death [84]. In addition to this, a recent cross-sectional study also highlighted that older adults who followed the MD and were physically active had positive beliefs regarding their physical health [85].

In accordance with our study, a recent study in Sweden documented that older persons who followed the MD also had higher physical activity levels [86]. In contrast, Foscolou et al. found similar physical activity levels in people with varying levels of MD adherence; however, this study included both the middle-aged and older-aged Greek population, which may influence the final findings concerning the individual life stage of older adults [8]. Regarding diet quality and physical activity levels, Japanese people over 85 years old that had a higher diet quality showed better physical activity levels [87]. Similarly, in Polish adults, sedentary behaviors correlated with lower diet quality [88].

As the global population ages, the promotion of a healthy lifestyle is an important strategy for healthy ageing. In this context, the promotion of healthy dietary patterns, such as the MD, and physical activity is a cost-effective way to improve health outcomes and promote healthy ageing [7,89,90]. Furthermore, taking into consideration the role of sleep in human health, the promotion of MD and physical activity can be recommended as means of increasing sleep quality in the age group of older adults. Evidently, the barriers to the implementation of these strategies should be considered, and we should focus on resolving these barriers accordingly [91]. Quality of life can be improved via different methods, including physical activity [92], a healthy dietary pattern [53], and a better sleep quality [91]. In the MD, in addition to the combination of foods, cultural and lifestyle elements are included in terms of selection, processing, and consumption, for example, through the prioritization of fresh, local, and seasonal foods, culinary activities and socializing at meals, regular physical activity, rest in the form of a nap, and a whole manner of living that is part of the cultural heritage of Mediterranean countries.

Several substantial studies have supported the holistic beneficial effect of MD on human health and wellbeing. Moderate or high MD adherence was associated with greater diabetes-specific quality of life in 258 participants with type 1 diabetes mellitus [93]. In a two-arm, two-site clinical trial, short-term MD compliance was effective in improving some microvascular physiological properties and quality of life domains, suggesting also that further improvement could be obtained in individuals with long-term MD compliance [94]. In a prospective, cross-sectional, case–control study conducted on 100 children and adolescents with irritable bowel syndrome (IBS), MD adherence was associated with considerable improvement in IBS scores, including participants’ quality of life [95]. In a 12-weeks, parallel-group, open-label, randomized, controlled trial conducted on 72 young males (18–25 years), an MD intervention resulted in considerable reductions in depression scores and increases in quality-of-life scores compared with befriending [96]. An observational study in Southern Italy on 407 persons adopting the MD showed that the extent of the food supply chain may exert a crucial impact on the risk of metabolic syndrome [97]. In addition, the MD exerted a substantial and beneficial impact on the quality of life and disease symptom scores in 475 individuals with multiple sclerosis [98]. An 18-month MD supplemented with plant-based polyphenols and lower levels of red and processed meat was found to be a promising intervention in promoting visceral adiposity regression in a randomized controlled clinical trial of 294 participants [99].

Interestingly, MD compliance was positively associated with quality of life and study time and negatively with TV and mobile phone usage among adolescents and medical students in the pre-lockdown period [100]. Moreover, greater MD compliance was associated with less perceived hardship and higher happiness and quality of life during lockdown [100]. In a longitudinal, single-center study conducted on 912 individuals with atrial fibrillation receiving vitamin K antagonists, gut-derived lipopolysaccharide levels were predictive of major adverse cardiovascular events, and were negatively affected by high MD compliance [101]. In a systematic review, healthy nutritional patterns were associated with favorable self-reported health and quality of life in one or more domains in older adults, and compliance to healthy dietary patterns such as the MD was considerably associated with improvement in at least one of the quality-of-life domains [16]. A greater adherence at baseline to an MD was also associated with a lower risk of mortality in older adults during a 20-year follow-up of 642 participants aged ≥ 65 years [102].

The present study has certain strengths, as it was performed on a quite representative population of older adults from various geographically diverse Greek regions, involving both urban, rural and island areas. It is one of the few studies that investigates the interrelationships between MD, physical activity levels, sleep quality and health-related quality of life in this age group in Greece. Moreover, we utilized the well-recognized PSQI questionnaire to reliably evaluate sleep quality. A simple randomization method was applied, which maintains complete randomness of the assignment of a participant to a particular group. In large-scale clinical research such as the current study, this method can be trusted to generate similar numbers of participants among groups. An almost equal number of male and female participants were included in the study population to avoid sex effects. Moreover, the study population was carefully selected to include an equal representation of all age groups beyond 65 years old. We further performed a statistical analysis by stratifying by participants’ sex and age, but we did not find any significant difference from the whole analysis (data not shown).

However, the interpretation of the present findings should be made with some limitations in mind. The cross-sectional design of the present survey limits the possibility for etiological conclusions, and exhibits the potential of recall biases especially for self-reported questions. Furthermore, although a comprehensive approach for confounding adjustment was performed, we recognize the probability of undetermined confounders. Although we have applied adjustment for age, gender, education, financial status, smoking habits, and living status, there is still the possibility that residual confounding may affect our findings. On the other hand, the fact that no conclusions about causality can be obtained due to the cross-sectional design of our study should be emphasized. Drug prescriptions are another potential confounding factor that should be taken into consideration in future studies.

The study population was enough large and contains Caucasian older adults who live in 14 geographically different urban, rural and island areas, and therefore its representativeness may be considered adequate. Hence, our findings may be generalizable outside the Greek population in other Caucasian populations of other ethnicities. However, future studies need to be conducted on other ethnicities, which may have several differences regarding genetic background, lifestyle factors, sociodemographic characteristics and nutritional habits compared to the Caucasian ethnicity.

## 5. Conclusions

Dietary patterns affect several fields of human health and well-being throughout life. Adherence to a healthy dietary pattern such as the MD has been related with favorable sleep quality, quality of life and physical activity levels, as all these factors are interconnected and important for successful ageing in Greek older adults. Strategies and public health policies that could promote MD adherence and physical activity for older populations may also facilitate sleep and quality of life, thus impacting overall wellbeing in this age group, which comprises a large proportion of the Greek population. Future large-scale, international, multicenter, population-based, epidemiological studies with samples of different ethnicities from geographically diverse areas of other countries worldwide, including urban, rural and island regions, are essential for more reliable conclusions to be drawn in older populations.

## Figures and Tables

**Table 1:** Association between Mediterranean diet adherence and participants’ sociodemographic parameters, anthropometric measures, HRQOL, IPAQ and PSQI categories.

Parameters, *n* = 3254	Mediterranean Diet Adherence				
	Very Low (25.8%)	Low (25.6%)	Moderate (24.8%)	High (23.8%)	*p*-Value
Age (yrs ± SD)	77.1 ± 8.5	74.7 ± 7.9	74.5 ± 8.8	72.5 ± 7.6	*p* < 0.0001
Sex (*n*, %)					*p* < 0.0001
Female	358 (42.6)	404 (48.6)	414 (51.4)	504 (64.9)	
Male	482 (57.4)	428 (51.4)	392 (48.6)	272 (35.1)	
Body mass index	27.7 ± 4.0	27.6 ± 3.8	27.4 ± 4.2	26.9 ± 4.3	*p* = 0.0047
Mid arm circumference (*n*, %)					*p* = 0.0008
<22 cm	278 (33.1)	232 (27.9)	216 (26.8)	115 (14.8)	
≥22 cm	562 (66.9)	600 (72.1)	590 (73.2)	661 (85.2)	
Calf circumference (*n*, %)					*p* = 0.0011
<31 cm	362 (43.1)	332 (39.9)	286 (35.5)	178 (22.9)	
≥31 cm	478 (56.9)	500 (60.1)	520 (64.5)	598 (77.1)	
Educational level (yrs ± SD)	7.0 ± 2.2	7.1 ± 2.7	7.3 ± 3.3	7.7 ± 3.0	*p* = 0.0026
Financial status (*n*, %)					*p* = 0.0005
Low	414 (49.3)	395 (47.5)	307 (38.1)	268 (34.5)	
Medium	377 (44.9)	371 (44.6)	272 (33.7)	222 (28.6)	
High	49 (5.8)	66 (7.9)	227 (28.2)	286 (36.9)	
Living status (*n*, %)					*p* = 0.0016
Alone	403 (48.0)	372 (44.7)	242 (30.0)	170 (21.9)	
With others	437 (52.0)	460 (52.3)	564 (70.0)	606 (78.1)	
Smoking habits (*n*, %)					*p* = 0.0031
Never smokers	683 (81.3)	691 (83.0)	542 (67.2)	513 (66.1)	
Smokers	157 (18.7)	141 (17.0)	264 (32.8)	263 (33.9)	
HRQOL (*n*, %)					*p* < 0.0001
Very low	273 (32.5)	256 (30.8)	163 (20.2)	124 (16.0)	
Low	251 (30.0)	246 (29.6)	154 (19.1)	130 (16.7)	
Moderate	158 (18.8)	180 (21.6)	258 (32.0)	256 (33.0)	
High	158 (18.8)	150 (18.0)	231 (28.7)	266 (34.3)	
IPAQ (*n*, %)					*p* < 0.0001
Low	435 (51.8)	408 (49.0)	281 (34.9)	206 (26.5)	
Moderate	389 (46.3)	360 (43.3)	232 (28.8)	168 (21.7)	
High	16 (1.9)	64 (7.7)	293 (36.3)	402 (51.8)	
PSQI (*n*, %)					*p* < 0.0001
Inadequate	312 (62.9)	461 (55.4)	233 (28.9)	222 (28.6)	
Adequate	249 (37.1)	371 (44.6)	573 (71.1)	554 (71.4)	

**Table 2:** Multiple logistic regression analysis for Mediterranean diet adherence after adjustment for participants’ age, sex, body mass index (BMI), mid arm and calf circumference, educational level, financial and living status, smoking habits, health-related quality of life (HRQOL), international physical activity questionnaire (IPAQ) levels and Pittsburgh sleep quality index (PSQI).

Parameters	OR (95% CI)	*p*-Value
Age		0.1173
Below mean value	1.0	
Over mean value	0.93 (0.48–1.51)	
Sex		0.0032
Male	1.0	
Female	1.36 (1.02–1.68)	
BMI		0.2657
Normal	1.0	
Overweight or obese	0.87 (0.36–1.49)	
Mid arm circumference		0.2895
<22 cm	1.0	
≥22 cm	1.22 (0.70–1.73)	
Calf circumference		0.1246
<31 cm	1.0	
≥31 cm	1.15 (0.77–1.54)	
Educational level		0.0812
Below mean value	1.0	
Over mean value	1.11 (0.58–1.63)	
Economic status		0.2301
Low or medium	1.0	
High	1.17 (0.73–1.60)	
Living status		0.0375
Alone	1.0	
With others	1.24 (0.81–1.76)	
Smoking habits		0.0763
No	1.0	
Yes	0.83 (0.42–1.30)	
HRQOL		0.0008
Very low or low	1.0	
Moderate or high	2.31 (2.06–2.68)	
IPAQ		0.0141
Low	1.0	
Moderate or high	1.89 (1.47–2.35)	
PSQI		0.0018
Inadequate	1.0	
Adequate	2.11 (1.79–2.44)	

## Data Availability

All of the data is contained within the article.
